# Diversity of Beetle Genes Encoding Novel Plant Cell Wall Degrading Enzymes

**DOI:** 10.1371/journal.pone.0015635

**Published:** 2010-12-17

**Authors:** Yannick Pauchet, Paul Wilkinson, Ritika Chauhan, Richard H. ffrench-Constant

**Affiliations:** 1 Biosciences, University of Exeter, Penryn, United Kingdom; 2 Department of Entomology, Max Planck Institute for Chemical Ecology, Jena, Germany; St Georges University of London, United Kingdom

## Abstract

Plant cell walls are a heterogeneous mixture of polysaccharides and proteins that require a range of different enzymes to degrade them. Plant cell walls are also the primary source of cellulose, the most abundant and useful biopolymer on the planet. Plant cell wall degrading enzymes (PCWDEs) are therefore important in a wide range of biotechnological processes from the production of biofuels and food to waste processing. However, despite the fact that the last common ancestor of all deuterostomes was inferred to be able to digest, or even synthesize, cellulose using endogenous genes, all model insects whose complete genomes have been sequenced lack genes encoding such enzymes. To establish if the apparent “disappearance” of PCWDEs from insects is simply a sampling problem, we used 454 mediated pyrosequencing to scan the gut transcriptomes of beetles that feed on a variety of plant derived diets. By sequencing the transcriptome of five beetles, and surveying publicly available ESTs, we describe 167 new beetle PCWDEs belonging to eight different enzyme families. This survey proves that these enzymes are not only present in non-model insects but that the multigene families that encode them are apparently undergoing complex birth-death dynamics. This reinforces the observation that insects themselves, and not just their microbial symbionts, are a rich source of PCWDEs. Further it emphasises that the apparent absence of genes encoding PCWDEs from model organisms is indeed simply a sampling artefact. Given the huge diversity of beetles alive today, and the diversity of their lifestyles and diets, we predict that beetle guts will emerge as an important new source of enzymes for use in biotechnology.

## Introduction

Plant cell walls are comprised of a mixture of complex polysaccharides and proteins which provide to the plant structural support as well as defence against pathogens. The primary cell walls is composed of two polysaccharide networks, one made from cellulose and hemicellulose, and the pectins [1]. Some microorganisms have become very effective in utilizing plant cell walls as a source of nutrients for their development making them efficient plant pathogens. These plant pathogenic bacteria and fungi secrete an impressive array of polysaccharide degrading enzymes, referred to here as plant cell wall degrading enzymes or PCWDEs. Among these, polygalacturonases, pectin methylesterases and pectin lyases degrade the pectin network, whereas various endoglucanases target the cellulose/hemicellulose network [2].

Much of the current literature emphasises the role of the gut microflora and symbiotic microbes in the breakdown of plant cell walls [3], [4], [5] and, in relation to insects, this view has been apparently reinforced by the apparent absence of genes encoding PCWDEs from model genomes such as those of the Red flour beetle *Tribolium castaneum* [6] and the silkworm *Bombyx mori* [7]. However, recently, there is a growing body of evidence that genes encoding enzymes from these families are indeed distributed in the genomes of a wide range of invertebrates including insects [8]. The first endogenous cellulase gene, encoding a functional enzyme from the glycoside hydrolase family 9 (GH9), was described in the termite *Reticulitermes speratus* [9], [10]. Since then, genes encoding putative GH9 enzymes have also been found in other insects [8]. More recently genes encoding cellulases from GH5 and GH45 families have been described in several longicorn beetles (family Cerambycidae) [11], [12], [13], [14] and in the mustard leaf beetle *Phaedon cochleariae* [15]. In addition beetle pectolytic enzymes have also been described, such as an endopolygalacturonase (GH28) and a pectin methylesterase (carbohydrate esterase family 8, CE8) from the Rice weevil *Sitophilus oryzae* [16], [17] and a endopolygalacturonase from the Mustard leaf beetle [15]. To increase the discovery rate of genes encoding beetle PCWDEs, we have recently adopted the 454-mediated pyrosequencing of beetle midguts as a standard sampling technique and in an initial study we have used this technique to describe the midgut transcriptome of the Poplar leaf beetle *Chrysomela tremulae*, which revealed several transcripts encoding a variety of PCWDEs [18].

In order to perform a more comprehensive survey of genes encoding beetle PCWDEs, here we extend this 454-mediated pyrosequencing approach to a wider range of beetles with different plant derived diets. Beetles are the most diverse group of animals on the planet and account for one fourth of all described species. Beetles appeared 285 million years ago and have since occupied nearly every available ecological niche, and it is this sustained diversification in a variety of plant associated niches, combined with the historically high survival of beetle lineages, that is thought to have led to their current success [19], [20]. Darwin himself was an enthusiastic beetle collector and he used them to illustrate many important biological concepts in his major works [21]. During this beetle niche radiation, two groups have become particularly effective at feeding on plants (herbivorous or xylophagous), the Chrysomeloidea (53,442 plant feeding species), which notably includes the leaf beetles and the longicorn beetles, and the Curculionoidea (59,340 plant feeders), which includes the weevils and the bark beetles, and as a result many have become important pests of crops, forests and stored products [20].

With the advent of next generation sequencing (NGS) it has become easier to look for PCWDEs in previously unexplored niches. We wanted to examine if the apparent absence of genes encoding PCWDEs from the genomes of model organisms was indeed a sampling artefact, and to test if other groups of insects, especially beetles, have in fact maintained genes encoding these enzymes from the last common ancestor of all deuterostomes [22], [23]. To address that, we targeted the ‘digestive transcriptome’ of beetle species with different diets in order to maximize our chances of recovering novel groups of PCWDEs. We generated normalized cDNA libraries from the midgut of each species and sequenced each library on a full plate of a Roche 454 pyrosequencer. We surveyed the resulting assembled ESTs for the presence of transcripts encoding PCWDEs. We also examined previously characterized enzymes, as well as collections of publicly available beetle ESTs. Using these combined datasets, here we describe a total of 167 novel enzymes from eight different families of PCWDEs, showing that beetles themselves can indeed breakdown the complex polysaccharides shaping plant cell walls, and may therefore represent a large new reservoir of PCWDEs for use in biotechnology.

## Results

### The diversity of beetle PCWDEs

To test whether genes encoding PCWDEs are indeed widely spread in phytophagous beetles, we pyrosequenced (454, Roche) cDNA libraries generated from five species of beetles, including the one from *C. tremulae* [18]. First we assessed the potential level of contamination of our EST datasets by eukaryotic organisms present in the gut flora or being potential endosymbionts. To do this we Blast searched a set of ‘reference genes’ known to be single copy genes, present in all eukaryotes and usually expressed at high levels, specifically we searched for homologs of the 79 ribosomal protein genes from *Tribolium* within our EST datasets. The only dataset for which we obtained two distinct hits for some of these 79 genes was that from the Green dock beetle. In this specific case, the second set of ribosomal protein transcripts comes from a yet undescribed microsporidial contaminant presumably present within the midgut tissue itself. It is therefore relatively facile to determine if a given EST dataset (particularly an insect gut dataset) is indeed contaminated by transcripts from another eukaryotic organism. Thus, again with reference to the Green dock beetle ∼6% of all the contigs we obtained matched one of the two microsporidian fully sequenced genomes from *Nosema ceranae* and *Encephalitozoon cuniculi*. In contrast, only a single set of ribosomal protein transcripts could be found in the other beetle EST datasets, indicating that no contamination by transcripts originating from another eukaryotic organism has occurred and that the ESTs presented are indeed from the beetles themselves. Finally, in order to confirm the hypothesis that the set of transcripts presented here were indeed beetle-derived we also compared their codon usage with the other genes present in the beetle EST datasets. The full codon usage table is presented in the supplementary materials (Table S1 and S2 in File S1) where beetle codon usages are also compared to those found in other insect associated microbes such as *Wolbachia*. In all cases the codon usage within PCWDE encoding genes was closer to that of the beetles (and *Tribolium*) themselves than to that of the microbes. Again consistent with the hypothesis that the ESTs (aside from those clearly matching microsporidia) are indeed beetle derived.

The five beetle species were chosen because they utilize different types of plant derived material as a food source and were thus predicted to display the greatest range and diversity of genes encoding PCWDEs (Table S3 in File S1). We then, in parallel, also performed a meta-analysis of all the publicly available coleopteran EST datasets present in the dbEST database at NCBI (Table S3 in File S1). These analyses revealed the presence of a total of 167 transcripts encoding PCWDEs which can be divided into eight strikingly large and diverse enzyme families or sub-families (Table S4 in File S1). These enzyme families can be classified into cellulolytic, pectolytic and hemicellulolytic (Table S4 in File S1). The largest gene family encodes polygalacturonases from the GH28 family. Transcripts for these enzymes ranged in number from as few as two transcripts, found in a limited EST dataset from the Coffee berry borer *Hypothenemus hampei*, to up to 19 different transcripts in the bark beetle *Dendroctonus ponderosae* (Table S4 in File S1).

### The predicted primary structure of beetle PCWDEs

The predicted primary structure of the beetle-derived PCWDEs is similar to other type of digestive enzymes found in insects but differs from microbial PCWDEs in several important respects. Beetle PCWDEs are composed of a catalytic domain and of an amino-terminal signal peptide, supporting the secretion of these enzymes from the midgut cells where they are produced to the gut lumen where they exert their biological function as digestive enzymes. Interestingly, all the beetle enzymes lack non-catalytic carbohydrate binding domains (CBM), widely found in microbial and plant derived PCWDEs, suggesting that their substrate binding properties may differ from previously characterized enzymes [24] (Figure S2 to S4 in File S1).

### Loss and gain of PCWDEs between different beetle groups

**Figure 1:**
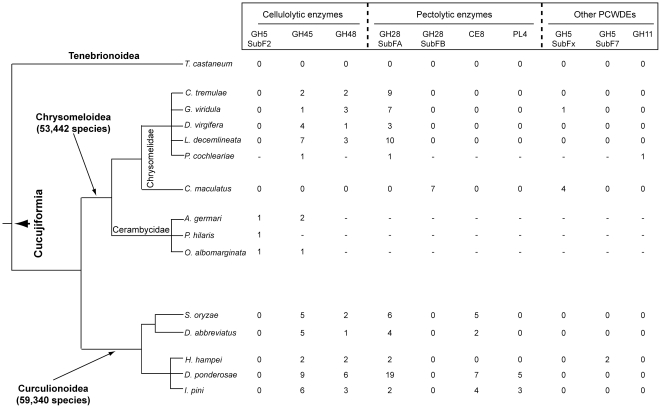
Summary of the beetle derived Plant cell wall degrading enzymes found in this study considered against the phylogeny of the beetles sampled. The various PCWDEs are classified in function of the specific polysaccharide they degrade (i.e. cellulose, pectin, hemicellulose). The nomenclature recommended by CAZy was used to further classify them. GH: Glycoside hydrolase; CE: Carbohydrate esterase; PL: Polysaccharide lyase. The number of transcripts found for each enzyme family after EST assembly and manual curation is also indicated.

The various PCWDEs families differ markedly in their apparent (sampled) distribution between beetles. When considered against the phylogeny of the beetles surveyed (Figure 1), the diversity of our beetle ESTs suggest that the large multigene families encoding beetle PCWDEs are undergoing complex ‘birth and death’ dynamics in the different taxonomic groups. For example, within the Chrysomelidae, beetles feeding on fresh plant material, such as the notorious Colorado potato beetle, carry several cellulolytic (GH45 and GH48) and pectolytic enzymes (GH28 subfamily A), whereas the bean beetle *Callosobruchus maculatus*, which feeds on pulses, has a totally different complement of enzymes, polygalacturonases from GH28 subfamily B and β-mannanases from a novel, unassigned, GH5 subfamily. Similarly, transcripts corresponding to cellulases from GH5 subfamily 2, characterized from three Cerambycidae species so far (Table S5 in File S1), could not be found in any of the EST datasets sampled (Figure 1). Also, we found transcripts encoding pectin methylesterases (CE8) only in species from the Curculionidae. Furthermore, within this very family, transcripts encoding rhamnogalacturonan lyase (PL4) could not be found in the Rice weevil or in *D. abbreviatus* and seem to be restricted to bark beetles only (subfamily Scolytinae). Finally, some enzymes, such as the xylanase (GH11) characterized from the Mustard leaf beetle and the β-mannanase (GH5 subfamily 7) from the Coffee berry borer, seem restricted to these two species alone (Figure 1 and Table S5 in File S1).

### Two distinct clades of GH28 enzymes in beetles

**Figure 2:**
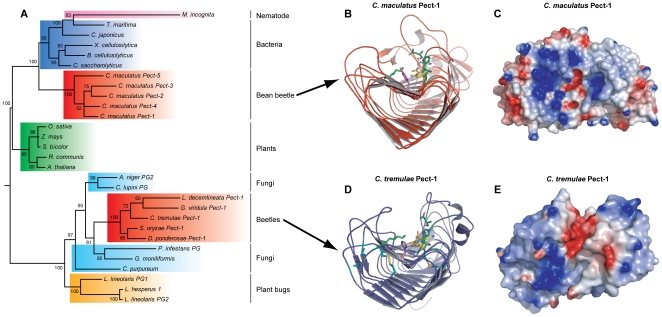
Beetle polygalacturonases (GH28) form two distinct clades, one more similar to those from bacteria and one more similar to those from fungi. **A**, A Bayesian inferred phylogeny is shown which compares the predicted amino acid sequences of the beetle GH28 enzymes described here with those known from bacteria, fungi, nematodes and plants. Posterior branch probabilities are shown and similar groupings were recovered using both Neighbor-joining and maximum likelihood based algorithms. **B**, Modelled cartoon view and **C**, Electrostatic map of *C. maculatus* Pect-1 enzyme oriented to view through the catalytic cleft. The proton donor (Asp^205^) is shown in white, and the catalytic nucleophile/base residues (Asp^184^ and Asp^206^) are shown in yellow. Conserved residues (Asn^182^, His^238^, Gly^239^ and Lys^271^) most likely implicated in substrate binding are shown in green, whereas the non conserved residue Tyr^269^ (instead of Arg) is shown in magenta. **D**, Modelled cartoon view and **E**, Electrostatic map of *C. tremulae* Pect-1 enzyme oriented to view through the catalytic cleft. The proton donor (Asp^185^) is shown in white, and the catalytic nucleophile/base residues (Asp^164^ and Asp^186^) are shown in yellow. Conserved residues (Asn^162^, His^207^, Gly^208^ and Lys^245^), most likely implicated in substrate binding, are in green, whereas the non conserved residue His^269^ (instead of Arg) is in magenta. The four conserved disulfide bridges are shown in cyan.

**Figure 3:**
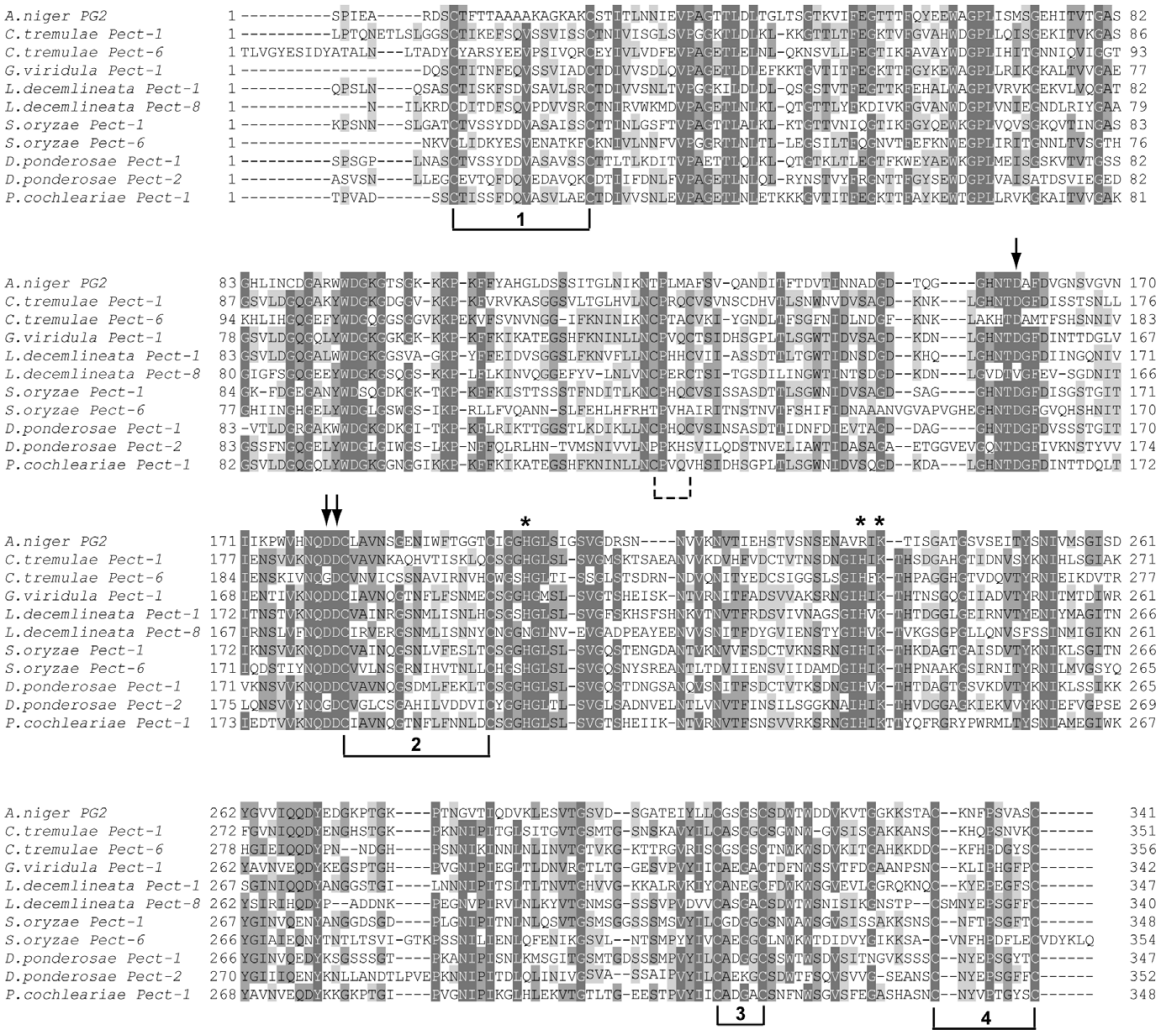
Predicted amino acid alignments of selected GH28 beetle enzymes. The amino acid sequence of the endopolygalacturonase II from *Aspergillus niger* (for which the crystal structure has been resolved) is used as a reference sequence. The catalytic residues, predicted from the *A. niger* sequence, are marked with arrows. Asp180 and Asp202 (numbering according to the *A. niger* sequence) act as the catalytic nucleophile/base, and Asp201 is the catalytic proton donor. The amino acid residue corresponding to Asp180 in *L. decemlineata* Pect-8 is a Val residue. The amino acid residue corresponding to Asp201 in *C. tremulae* Pect-6 and *D. ponderosae* Pect-2 is a Gly residue. Such changes may affect the catalytic abilities of the given enzymes. The four conserved disulfide bridges are numbered. An extra two Cys residues found in some sequences can form an extra disulfide bridge indicated by the dashed line.

A Bayesian inferred phylogenetic analysis of the catalytic domain of beetle derived GH28 enzymes together with their bacterial, fungal, plant, nematode and plant bug counterparts shows that they form two distinct clades (Figure 2a). The GH28 enzymes from the bean beetle *C. maculatus* are more closely related to bacterial derived enzymes, whereas the polygalacturonases derived from all the other beetles surveyed are more closely related to their fungal and plant bug counterparts. We have tentatively named these two distinct groups GH28 subgroup A and subgroup B, as no subgroups have currently been defined by CAZy (http://www.cazy.org) [25] for this enzyme family. Structural modelling of members of these two GH28 groups shows that they share a similar backbone made of parallel β-sheets (Figure 2b and 2d), but markedly differ by the presence of four strictly conserved disulfide bridges found only in enzymes from subgroup A (Figure 2d). Also, one of the active site residues implicated in substrate binding, an Arg in all characterized enzymes so far (Figure 3) [26], is replaced by an His (His^243^ in *C. tremulae* Pect-1) in all enzymes from subgroup A, and by aromatic residues, either a Tyr (Tyr^269^ in *C. maculatus* Pect-1) or a Phe, in enzymes from subgroup B (Figure 2b and 2d). These amino acid changes may reflect the adaptation of these enzymes to the physiological parameters characterising the digestive fluid present in the beetle gut lumen. Finally, these two groups of enzymes also show differences in the apparent accessibility of their respective catalytic clefts by the substrate, with *C. tremulae* Pect-1 having a relatively ‘open’ cleft compared to *C. maculatus* Pect-1 (Figure 2c and 2e).

An amino acid alignment of selected beetle derived polygalacturonases (Figure 3) revealed that some of the predicted proteins may have lost their catalytic activity due to the replacement of one Asp residue from their catalytic triad by a hydrophobic amino acid such as a Val residue in *L. decemlineata* Pect-8 or a Gly residue in both *C. tremulae* Pect-6 and *D. ponderosae* Pect-2. Also, this amino acid alignment suggests the possibility of an extra disulfide bridge, compared to the *A. niger* PGII taken as reference, due to the presence of two extra Cys residues in only some of the predicted proteins such as in *C. tremulae* Pect-1 and Pect-6 or in *G. viridula* Pect-1 (Figure 3). These extra Cys residues are completely missing, in *S. oryzae* Pect-6 or *D. ponderosae* Pect-2 for example, or only one is missing in *P. cochleariae* Pect-1.

### Two different classes of beetle GH45 enzymes

**Figure 4:**
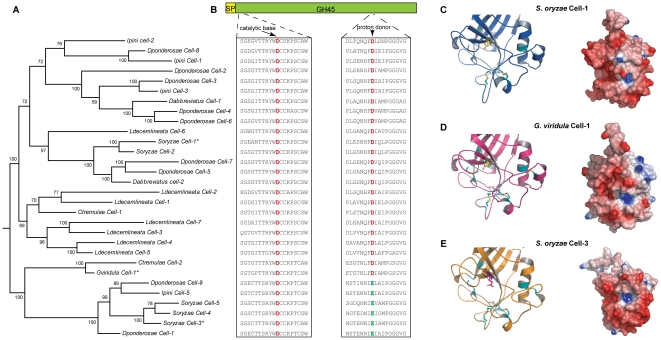
Phylogenetic and structural relationships of beetle-derived GH45 enzymes. **A**, Bayesian inferred phylogeny of the beetle GH45 enzymes surveyed. Posterior branch probabilities are shown and similar groupings were recovered using both Neighbor-joining and maximum likelihood based algorithms. The enzymes we modelled are indicated by an asterisk. **B**, Schematic representation of the primary structures of beetle derived beta-1,4-glucanases. An amino acid alignment of the regions surrounding both the proton donor and catalytic nucleophile/base for each sequence is shown underneath the primary structure. Note the distinct clade of beetle GH45s which carry a Glu (green) rather than Asp (red) as a putative proton donor. In contrast, the predicted catalytic nucleophile/base is conserved throughout all sequences surveyed. **C**, Modelled cartoon view and electrostatic map of *S. oryzae* Cell-1, which is part of the dominant clade of beetle beta-1,4-glucanases, shown in the same orientation. The catalytic nucleophile/base (Asp^23^) is shown in white, and the catalytic proton donor (Asp^135^) is shown in yellow. **D**, Modelled cartoon view and electrostatic map of *G. viridula* Cell-1 shown in the same orientation. The catalytic nucleophile/base (Asp^24^) is shown in white and the catalytic proton donor (Asp^136^) is shown in yellow. **E**, Modelled cartoon view and electrostatic map of *S. oryzae* Cell-3 shown in the same orientation. The catalytic nucleophile/base (Asp^29^) is shown in white and the catalytic proton donor (Glu^146^) is shown in magenta. The six conserved disulfide bridges are indicated in cyan.

Although harbouring fewer members than the GH28 family, the beetle GH45 family (β-1,4-glucanases) is also relatively large showing as few as a single GH45-encoding transcript in the Green dock beetle *G. viridula* and up to nine different enzymes in the Mountain pine beetle *D. ponderosae* (Figure 1). A Bayesian inferred phylogeny of all beetle encoded GH45 enzymes (Figure 4a) shows two distinct enzyme groups which differ in their potential proton donor residues with a Asp being replaced by a Glu residue (Figure 4b), and further structural modelling predicts that replacement of these critical residues may alter the accessibility of the active site potentially resulting in a dramatic change in the enzymatic properties of these proteins (Figure 4c-e). Interestingly, GH45 enzymes harbouring a Glu residue as catalytic proton donor seem restricted to species from the family Curculionidae. Taken together, these novel enzyme attributes suggest that beetle PCWDEs may represent interesting alternatives to microbial PCWDEs for use in biotechnology and suggest that the sequencing of a wider range of beetles will provide a diverse source of such enzymes.

## Discussion

Here we show that not only enzymes able to degrade the diverse polysaccharides shaping plant cell walls are indeed present in a range of phytophagous beetles, but also that these enzymes have diversified and are now part of large multigene families. Codon usage analysis and matches with known microbial endosymbionts suggest that microbial transcripts are relatively easy to differentiate from those of the beetle itself and support the assumption that the ESTs are of beetle origin. Further work to look at their relative locations in the beetle genome (large insert libraries of beetle genomic DNA) are now underway. Importantly, no homologs of the PCWDEs we describe here are found in the complete genome of the red flour beetle *Tribolium castaneum*, the only coleopteran genome sequenced to date [24], although a gene encoding a putative cellulase from the GH9 family is present in this species [8]. Our analysis also clearly demonstrates that these enzyme families have undergone complex birth and death dynamics even in closely related species.

Interestingly, the apparent diversity of genes encoding beetle PCWDEs also suggests that members of these multigene families may have evolved new function through either sub- or neo-functionalisation. For example, modification of amino acid residues of the catalytic triad from Asp to hydrophobic amino acids in some members of the GH28 family, suggesting a loss of activity, is very similar to what has already been described for the serine proteinase enzyme family in Lepidoptera. Some members of this family, although lacking enzymatic activity due to replacement of critical amino acids of their catalytic triad, have been suggested to play a very important role in the digestive process by efficiently binding to plant derived serine proteinase inhibitors therefore preventing them to inhibit active enzymes [27]. Further, despite having classified the beetle GH48 enzymes as putative cellobiosidases, according to their high degree of similarity with their bacterial counterparts with known cellobiosidase activity (Figure S1 in File S1) and to an enzyme isolated from the Black vine weevil *Otiorhynchus sulcatus* annotated as a cellulose 1,4-β-cellobiosidase (Table S5 in File S1), a recent report suggests that these enzymes are important in diapause termination in beetles by virtue of having evolved to degrade chitin rather than cellulose [28].

The striking amplification and diversification in the number of genes encoding beetle PCWDEs can potentially be related to the presence, in plants, of inhibitors of PCWDEs, which have been implicated in defence against phytopathogens, especially fungi [2], [29]. The presence of such inhibitors in plants may have led to an evolutionary ‘arms race’ whereby beetles had to diversify their PCWDEs arsenal to adapt to the inhibitors synthesized by their respective host plants, potentially leading to inhibitor-insensitive enzymes [27]. This may present a significant potential advantage of beetle derived PCWDEs over those from microbes for their use in biotechnological processes, as some current limitations of microbial PCWDEs are related to their enzymatic stability and susceptibility to inhibitors or plant by-products [30].

The emphasis in the current study on transcripts encoding PCWDEs transcripts from the two beetle groups the Chrysomeloidea and Curculionoidea highlights their efficiency as phytophagous and xylophagous insects. However, there is a clear bias in the coleopteran derived ESTs present in dbEST at NCBI towards species from these two superfamilies. A complete overview of the distribution of PCWDEs within the Coleoptera will require generating and analyzing datasets from species coming from other clades known to contain living plant feeders such as the Melolonthinae (Chafers), the Byturidae (Fruitworm beetles) and the Epilachninae (Plant-eating Lady beetles) [20].

In conclusion, we have shown, via pyrosequencing of midgut RNA from a selected range of beetles, that beetle guts themselves (as well as their gut microflora) are indeed a diverse source of PCWDEs. This finding is supported by another recently published study using a similar approach to mine cellulases from the digestive system of a wood boring marine isopod [31], suggesting that the arthropods as a whole may show a similar diversity in PCWDEs. Despite the obvious limitations in the EST based sampling of transcriptomes from specific insect tissues (versus complete genome sequencing), the shear diversity of beetle PCWDEs revealed here begins to suggest that the different families of genes encoding beetle PCWDEs are undergoing dramatic birth-death dynamics as beetles have radiated to exploit different niches and food sources. Previously, the success of beetles as a group has been attributed to their exploitation of a range of different ecological niches and their ability to persist historically within these niches [20]. In turn, this study also suggests that the wealth of PCWDEs found in beetles reflects their repeated exploitation of different plant derived diets in evolutionary time and that the search for different members of such multigene families in other beetle groups may be equally productive. Clearly a full understanding of the complex biology of the Coleoptera as a whole cannot rely on the genome of *T. castaneum* alone and sequencing the genomes of other key beetles will be necessary to our further understanding of beetle biology.

## Materials and Methods

### Midgut cDNA library preparation and sequencing

Total RNA were isolated from larval midgut of *G. viridula* and *L. decemlineata*, adult midgut from *S. oryzae*, and whole *C. maculatus* larvae according to [18]. Full-length, enriched, cDNAs were generated from 2 µg of total RNA using the SMART PCR cDNA synthesis kit (BD Clontech) following the manufacturer's protocol. To prevent over-representation of the most abundant transcripts, the resulting double-stranded cDNAs were normalized using the Kamchatka crab duplex-specific nuclease method (Trimmer cDNA normalization kit, Evrogen) [32]. For 454 pyrosequencing, a cDNA aliquot of each library was sent to the Advanced Genomics facility at the University of Liverpool (http://www.liv.ac.uk/agf). A single full plate run of 454 Titanium (Roche Applied Science) was performed per library using 3 µg of normalized cDNAs processed by the “shotgun” method. Trimming and assembly of the raw nucleotide sequences was achieved using our in house pipeline called ‘*est2assembly*’ [33]. All 454 derived sequences have been submitted to the Short Read Archive (SRA) database at NCBI (SRX017237-SRX017241).

### Assembly of beetle ESTs from public databases

EST datasets from each species were retrieved from the dbEST public database (NCBI) as FASTA files. These datasets were then assembled using the SeqMan Pro assembler of the Lasergene software package v8.0.2 (DNASTAR, Madison USA) with the following program parameters: match size, 50bp; minimum match percentage, 80%; minimum sequence length, 40 bp; gap length penalty, 0.70 and maximum mismatch end bases, 15.

### Blast homology searches and sequence annotation

Homology searches (BLASTX and BLASTN) of unique sequences and functional annotation by gene ontology terms (GO; www.geneontology.org), InterPro terms (InterProScan, EBI), enzyme classification codes (EC), and metabolic pathways (KEGG, Kyoto Encyclopedia of Genes and Genomes) were determined using the Blast2GO software suite v2.4.2 (www.blast2go.org) [34]. Homology searches were performed remotely on the NCBI server through QBLAST. Sequences were searched against an NCBI non redundant (nr) protein database via BLASTx using an E-value cutoff of 10^−6^ and selecting predicted polypeptides of a minimum length of 10 amino acids. For gene ontology mapping, the program extracts the GO terms associated with homologies identified with NCBI's QBLAST and returns a list of GO annotations represented as hierarchical categories of increasing specificity. Blast2GO allows the selection of a significance level for the False Discovery Rate (FDR) which was used as a cut-off at the 0.05% probability level. Then, GO terms were modulated using the annotation augmentation tool ANNEX [35] followed by GOSlim. GOSlim consists of a subset of the gene ontology vocabulary encompassing key ontological terms and a mapping function between the full GO and the GOSlim. Here, we used the “generic” GOSlim mapping (goslim_generic.obo) available in Blast2GO. Enzyme classification codes and KEGG metabolic pathway annotations are generated from the direct mapping of GO terms to their enzyme code equivalents. Finally, InterPro searches were performed remotely from Blast2GO to the InterProEBI web server. The default settings of Blast2GO were used in every annotation step.

### Full length sequencing and manual curation of cDNAs

Contigs corresponding to sequences of interest were retrieved, re-assembled one by one using the SeqMan Pro assembler, and manually curated to correct potential assembly errors. cDNA sequences derived from *C. tremulae*, *G. viridula*, *L. decemlineata*, *S. oryzae* and *C. maculatus*, encoding only a partial open reading frame (ORF), were used to design specific primer pairs to perform 5′- and 3′-Rapid Amplification of cDNA ends (RACE) PCRs. For these we used the SMART RACE cDNA Amplification Kit (BD Clontech) according to the manufacturer's instructions. All cDNA sequences encoding the complete ORF were annotated and submitted to Genbank under accession numbers HM175741 to HM175859.

### Phylogenetic analyses

The analyses were performed on the Phylogeny.fr [36] platform. Sequences were aligned with MUSCLE (v3.7) configured for highest accuracy (MUSCLE with default settings). After alignment, ambiguous regions (i.e. containing gaps and/or poorly aligned) were removed with Gblocks (v0.91b) using the following parameters: minimum length of a block after gap cleaning: 5; positions with a gap in less than 50% of the sequences were selected in the final alignment if they were within an appropriate block; all segments with contiguous nonconserved positions bigger than 8 were rejected; minimum number of sequences for a flank position: 55%. The phylogenetic tree was constructed using the bayesian inference method implemented in the MrBayes program (v3.1.2). The number of substitution types was fixed to 6. The Poisson model was used for amino acid substitution, while rates variation across sites was fixed to “invgamma”. Four Markov Chain Monte Carlo (MCMC) chains were run for 100,000 generations, sampling every 10 generations, with the first 250 sampled trees discarded as “burn-in”. Finally, a 50% majority rule consensus tree was constructed. Graphical representation and edition of the phylogenetic tree were performed with TreeDyn (v198.3).

### Enzyme 3D modelling

Web based tools FUGUE [37] and PHYRE [38] were used to find the appropriate templates for each protein. The query sequences were also BLAST searched against the Protein Data Bank (PDB) to search for homologues. In order to increase the model accuracy more than one template was used for modelling each of the five PCWDEs. Multiple alignment of the protein of interest and the selected template sequences were executed using ClustalW [39]. The multiple alignments of the query sequence and protein sequences were also analysed using JOY [40], which produces formatted alignments highlighting unique patterns of amino acids substitutions in various environments, thus helping to identify misaligned regions or residues that play important structural role. Modeller 9v8 program [41] was used to derive the three-dimensional models of each PCWDE. Ten alternative preliminary models were generated using standard settings and were evaluated using a web based structural analysis and verification server (http://nihserver.mbi.ucla.edu/SAVES_3/). The model with the lowest energy and the lowest restraint violation was selected as the target model for further analysis. Electrostatic potential maps were calculated using Delphi software [42] using the default parameters. Visualisation of the models was done using Pymol (http://pymol.sourceforge.net/).

## Supporting Information

File S1 **Codon usage of PCWDEs characterised in this study compared to those obtained from whole beetle transcriptomes, model insects and representatives of microbes (Table S1 and S2).** Summary statistics for beetle EST datasets (Table S3). Families of beetle plant cell wall degrading enzymes identified in coleopteran-derived EST datasets (Table S4). cDNAs encoding beetle plant cell wall degrading enzymes identified from public databases (Table S5). Predicted amino acid alignment of PCWDEs sequences described in this study (Figure S1 to S4).
(DOC)
