# Supplementary material for: Diversity of Beetle Genes Encoding Novel Plant Cell Wall Degrading Enzymes
Source: PLoS One. 2010 Dec 17;5(12):e15635. doi: 10.1371/journal.pone.0015635 (PMC3003705; doi:10.1371/journal.pone.0015635)
Supplement: File S1 — Codon usage of PCWDEs characterised in this study compared to those obtained from whole beetle transcriptomes, model insects and representatives of microbes (Table S1 and S2). Summary statistics for beetle EST datasets (Table S3). Families of beetle plant cell wall degrading enzymes identified in coleopteran-derived EST datasets (Table S4). cDNAs encoding beetle plant cell wall degrading enzymes identified from public databases (Table S5). Predicted amino acid alignment of PCWDEs sequences described in this study (Figure S1 to S4). (DOC) [file pone.0015635.s001.doc]

**Table S1.** Codon usage of PCWDEs characterised in this study compared to those obtained from whole beetle transcriptomes, model insects (*T. castaneum* and *D. melanogaster*) and representatives of microbes (*Nocema Bombycis*, *Saccharomyces cerevisiae* and *Wolbachia*). The preferred codon(s) for each amino acid is highlighted in red.

| **#Codon (amino acid)** | **PCWDEs** | **C.maculatus** | **C.tremulae** | **G.viridula** | **L.decemlineata** | **S.oryzae** | **T.castaneum** | **D.melanogaster** | **Nosema** | **S.cerevisiae** | **Wolbachia** |
| --- | --- | --- | --- | --- | --- | --- | --- | --- | --- | --- | --- |
| GCA (A) | **0,287** | **0,346** | **0,352** | **0,361** | **0,348** | **0,322** | **0,329** | **0,314** | 0,27 | **0,29** | **0,387** |
| GCC (A) | 0,236 | 0,201 | 0,219 | 0,204 | 0,211 | 0,215 | 0,2 | 0,234 | 0,22 | 0,22 | 0,146 |
| GCG (A) | 0,161 | 0,132 | 0,116 | 0,128 | 0,127 | 0,145 | 0,197 | 0,187 | 0,15 | 0,11 | 0,11 |
| GCT (A) | **0,316** | **0,321** | **0,312** | **0,306** | **0,314** | **0,318** | **0,274** | **0,265** | **0,36** | **0,38** | **0,357** |
| TGC (C) | 0,414 | 0,392 | 0,38 | 0,362 | 0,376 | 0,351 | 0,361 | 0,483 | 0,26 | 0,37 | **0,503** |
| TGT (C) | **0,586** | **0,608** | **0,62** | **0,638** | **0,624** | **0,649** | **0,639** | **0,517** | **0,74** | **0,63** | 0,497 |
| GAC (D) | 0,433 | 0,381 | 0,346 | 0,347 | 0,366 | 0,373 | 0,407 | 0,416 | 0,26 | 0,35 | 0,273 |
| GAT (D) | **0,567** | **0,619** | **0,654** | **0,653** | **0,634** | **0,627** | **0,593** | **0,584** | **0,74** | **0,65** | **0,727** |
| GAA (E) | **0,65** | **0,66** | **0,68** | **0,682** | **0,678** | **0,689** | **0,698** | **0,611** | **0,72** | **0,7** | **0,668** |
| GAG (E) | 0,35 | 0,34 | 0,32 | 0,318 | 0,322 | 0,311 | 0,302 | 0,389 | 0,28 | 0,3 | 0,332 |
| TTC (F) | 0,477 | 0,358 | 0,386 | 0,41 | 0,387 | 0,315 | 0,251 | 0,327 | 0,26 | 0,41 | 0,316 |
| TTT (F) | **0,523** | **0,642** | **0,614** | **0,59** | **0,613** | **0,685** | **0,749** | **0,673** | **0,74** | **0,59** | **0,684** |
| GGA (G) | **0,376** | **0,337** | **0,356** | **0,364** | **0,359** | **0,341** | **0,305** | **0,286** | **0,4** | 0,22 | **0,33** |
| GGC (G) | 0,198 | 0,203 | 0,185 | 0,185 | 0,184 | 0,197 | 0,213 | 0,273 | 0,12 | 0,19 | 0,201 |
| GGG (G) | 0,178 | 0,167 | 0,186 | 0,169 | 0,177 | 0,17 | 0,192 | 0,214 | 0,22 | 0,12 | 0,145 |
| GGT (G) | 0,248 | 0,292 | 0,274 | 0,282 | 0,28 | 0,292 | 0,29 | 0,227 | 0,17 | **0,47** | **0,324** |
| CAC (H) | 0,44 | 0,387 | 0,372 | 0,366 | 0,378 | 0,377 | 0,43 | 0,443 | 0,31 | 0,36 | 0,366 |
| CAT (H) | **0,56** | **0,613** | **0,628** | **0,634** | **0,622** | **0,623** | **0,57** | **0,557** | **0,69** | **0,64** | **0,634** |
| ATA (I) | 0,312 | 0,358 | 0,339 | 0,355 | 0,335 | 0,366 | 0,314 | 0,337 | 0,33 | 0,27 | 0,377 |
| ATC (I) | 0,315 | 0,244 | 0,24 | 0,24 | 0,241 | 0,207 | 0,171 | 0,222 | 0,16 | 0,26 | 0,213 |
| ATT (I) | **0,373** | **0,398** | **0,421** | **0,405** | **0,424** | **0,427** | **0,515** | **0,441** | **0,51** | **0,46** | **0,41** |
| AAA (K) | **0,612** | **0,665** | **0,674** | **0,676** | **0,683** | **0,712** | **0,963** | **0,819** | **0,69** | **0,58** | **0,667** |
| AAG (K) | 0,388 | 0,335 | 0,326 | 0,324 | 0,317 | 0,288 | 0,037 | 0,181 | 0,31 | 0,42 | 0,333 |
| CTA (L) | 0,134 | 0,13 | 0,12 | 0,127 | 0,115 | 0,127 | 0,107 | 0,104 | 0,11 | 0,14 | 0,136 |
| CTC (L) | 0,14 | 0,107 | 0,117 | 0,12 | 0,121 | 0,093 | 0,086 | 0,124 | 0,06 | 0,06 | 0,095 |
| CTG (L) | 0,202 | 0,15 | 0,146 | 0,148 | 0,147 | 0,125 | 0,094 | 0,151 | 0,07 | 0,11 | 0,11 |
| CTT (L) | 0,158 | 0,187 | 0,184 | 0,179 | 0,19 | 0,182 | 0,165 | 0,175 | 0,21 | 0,13 | 0,205 |
| TTA (L) | 0,161 | 0,207 | 0,195 | 0,188 | 0,196 | 0,27 | **0,325** | **0,223** | **0,38** | **0,28** | **0,259** |
| TTG (L) | **0,206** | **0,219** | **0,238** | **0,239** | **0,231** | **0,203** | 0,222 | **0,223** | 0,17 | **0,29** | 0,195 |
| ATG (M) | 1 | 1 | 1 | 1 | 1 | 1 | 1 | 1 | 1 | 1 | 1 |
| AAC (N) | 0,435 | 0,398 | 0,362 | 0,356 | 0,382 | 0,367 | 0,334 | 0,373 | 0,27 | 0,41 | 0,338 |
| AAT (N) | **0,565** | **0,602** | **0,638** | **0,644** | **0,618** | **0,633** | **0,666** | **0,627** | **0,73** | **0,59** | **0,662** |
| CCA (P) | **0,398** | **0,389** | **0,405** | **0,401** | **0,39** | **0,379** | **0,345** | **0,354** | **0,33** | **0,42** | **0,398** |
| CCC (P) | 0,188 | 0,167 | 0,186 | 0,167 | 0,178 | 0,171 | 0,191 | 0,213 | 0,13 | 0,15 | 0,152 |
| CCG (P) | 0,16 | 0,13 | 0,117 | 0,131 | 0,135 | 0,15 | 0,207 | 0,202 | 0,16 | 0,12 | 0,104 |
| CCT (P) | 0,255 | 0,314 | 0,291 | 0,301 | 0,297 | 0,3 | 0,257 | 0,231 | **0,38** | 0,31 | 0,346 |
| CAA (Q) | **0,629** | **0,591** | **0,616** | **0,619** | **0,615** | **0,62** | **0,702** | **0,598** | **0,69** | **0,69** | **0,644** |
| CAG (Q) | 0,371 | 0,409 | 0,384 | 0,381 | 0,385 | 0,38 | 0,298 | 0,402 | 0,31 | 0,31 | 0,356 |
| AGA (R) | **0,252** | **0,342** | **0,372** | **0,359** | **0,346** | **0,339** | **0,261** | **0,225** | **0,48** | **0,48** | **0,406** |
| AGG (R) | 0,219 | 0,208 | 0,211 | 0,19 | 0,198 | 0,186 | 0,144 | 0,16 | 0,24 | 0,21 | 0,211 |
| CGA (R) | 0,167 | 0,146 | 0,151 | 0,175 | 0,165 | 0,158 | 0,192 | 0,182 | 0,1 | 0,07 | 0,104 |
| CGC (R) | 0,087 | 0,087 | 0,071 | 0,073 | 0,073 | 0,084 | 0,117 | 0,151 | 0,04 | 0,06 | 0,093 |
| CGG (R) | 0,134 | 0,084 | 0,086 | 0,081 | 0,09 | 0,093 | 0,115 | 0,14 | 0,06 | 0,04 | 0,066 |
| CGT (R) | 0,141 | 0,134 | 0,109 | 0,122 | 0,128 | 0,14 | 0,17 | 0,142 | 0,08 | 0,14 | 0,12 |
|  |  |  |  |  |  |  |  |  |  |  |  |
|  |  |  |  |  |  |  |  |  |  |  |  |
| **#Codon (amino acid)** | **PCWDEs** | **C.maculatus** | **C.tremulae** | **G.viridula** | **L.decemlineata** | **S.oryzae** | **T.castaneum** | **D.melanogaster** | **Nosema** | **S.cerevisiae** | **Wolbachia** |
| AGC (S) | 0,147 | 0,14 | 0,12 | 0,108 | 0,114 | 0,123 | 0,119 | 0,169 | 0,07 | 0,11 | 0,168 |
| AGT (S) | 0,17 | 0,185 | 0,177 | 0,181 | 0,178 | 0,193 | 0,2 | 0,171 | 0,25 | 0,16 | 0,195 |
| TCA (S) | **0,25** | **0,228** | **0,253** | **0,261** | **0,243** | **0,21** | **0,225** | **0,187** | 0,21 | **0,21** | 0,221 |
| TCC (S) | 0,15 | 0,139 | 0,146 | 0,133 | 0,149 | 0,142 | 0,125 | 0,156 | 0,08 | 0,16 | 0,121 |
| TCG (S) | 0,111 | 0,094 | 0,09 | 0,107 | 0,102 | 0,106 | 0,14 | 0,143 | 0,09 | 0,1 | 0,06 |
| TCT (S) | 0,172 | 0,214 | 0,214 | 0,211 | 0,214 | **0,226** | 0,191 | 0,174 | **0,3** | **0,26** | **0,235** |
| ACA (T) | **0,306** | **0,367** | **0,37** | **0,381** | **0,36** | **0,361** | **0,365** | **0,347** | 0,32 | 0,3 | 0,322 |
| ACC (T) | 0,222 | 0,2 | 0,212 | 0,184 | 0,198 | 0,192 | 0,17 | 0,199 | 0,14 | 0,22 | 0,204 |
| ACG (T) | 0,186 | 0,142 | 0,124 | 0,133 | 0,134 | 0,149 | 0,178 | 0,179 | 0,19 | 0,14 | 0,115 |
| ACT (T) | 0,287 | 0,291 | 0,294 | 0,302 | 0,308 | 0,298 | 0,287 | 0,275 | **0,35** | **0,35** | **0,359** |
| GTA (V) | 0,236 | 0,273 | 0,255 | 0,269 | 0,254 | 0,28 | 0,245 | 0,225 | 0,33 | 0,21 | 0,317 |
| GTC (V) | 0,215 | 0,187 | 0,18 | 0,183 | 0,186 | 0,178 | 0,172 | 0,196 | 0,14 | 0,21 | 0,131 |
| GTG (V) | 0,23 | 0,213 | 0,224 | 0,222 | 0,205 | 0,186 | 0,205 | 0,257 | 0,18 | 0,19 | 0,201 |
| GTT (V) | **0,318** | **0,327** | **0,341** | **0,326** | **0,355** | **0,356** | **0,378** | **0,322** | **0,35** | **0,39** | **0,351** |
| TGG (W) | 1 | 1 | 1 | 1 | 1 | 1 | 1 | 1 | 1 | 1 | 1 |
| TAC (Y) | 0,464 | 0,378 | 0,341 | 0,346 | 0,356 | 0,348 | 0,348 | 0,351 | 0,38 | 0,44 | 0,335 |
| TAT (Y) | **0,536** | **0,622** | **0,659** | **0,654** | **0,644** | **0,652** | **0,652** | **0,649** | **0,62** | **0,56** | **0,665** |
| TAA (*) | 0,385 | **0,384** | 0,355 | 0,333 | 0,37 | **0,479** | **0,544** | **0,458** | **0,7** | **0,47** | **0,466** |
| TAG (*) | 0,193 | 0,243 | 0,221 | 0,226 | 0,216 | 0,226 | 0,179 | 0,212 | 0,1 | 0,23 | 0,241 |
| TGA (*) | **0,422** | 0,373 | **0,424** | **0,441** | **0,413** | 0,295 | 0,277 | 0,33 | 0,2 | 0,3 | 0,293 |

**Table S2.** Codon usage of PCWDEs characterised in this study compared to those obtained from whole beetle transcriptomes, model insects (*T. castaneum* and *D. melanogaster*) and representatives of microbes (*Nocema Bombycis*, *Saccharomyces cerevisiae* and *Wolbachia*). The data are expressed in relative synonymous codon usage (RSCU) values. RSCU values lower than 1 indicate that a codon is avoided and values higher than 1 indicates the given codon is preferred. A higher RSCU value indicates a higher preference for the particular codon. The preferred codon(s) for each amino acid is highlighted in red.

| **#Codon (amino acid)** | **PCWDEs** | **C.maculatus** | **C.tremulae** | **G.viridula** | **L.decemlineata** | **S.oryzae** | **T.castaneum** | **D.melanogaster** | **Nosema** | **S.cerevisiae** | **Wolbachia** |
| --- | --- | --- | --- | --- | --- | --- | --- | --- | --- | --- | --- |
| GCA (A) | **4** | **4** | **5** | **5** | **5** | **3** | **2** | **5** | 3 | 5 | **7** |
| GCC (A) | 2 | 2 | 1 | 1 | 1 | 1 | 1 | 3 | 2 | 3 | 1 |
| GCG (A) | 1 | 1 | 0 | 0 | 0 | 0 | 0 | 1 | 0 | 0 | 0 |
| GCT (A) | **4** | **4** | 3 | 3 | 3 | **3** | **2** | 4 | **7** | **10** | **7** |
| TGC (C) | 6 | 6 | 5 | 5 | 5 | 5 | 3 | 8 | 1 | 2 | **15** |
| TGT (C) | **17** | **17** | **18** | **20** | **19** | **19** | **13** | **17** | **112** | **8** | 7 |
| GAC (D) | 7 | 7 | 4 | 5 | 4 | 4 | 3 | 4 | 4 | 10 | 2 |
| GAT (D) | **19** | **19** | **18** | **19** | **17** | **16** | **9** | **13** | **38** | **37** | **16** |
| GAA (E) | **24** | **24** | **25** | **26** | **25** | **22** | **14** | **18** | **55** | **45** | **20** |
| GAG (E) | 6 | 6 | 4 | 4 | 4 | 3 | 2 | 5 | 7 | 6 | 3 |
| TTC (F) | 9 | 9 | 12 | 12 | 12 | 7 | 4 | 6 | 4 | 9 | 7 |
| TTT (F) | **21** | **21** | **38** | **36** | **40** | **46** | **43** | **37** | **40** | **26** | **45** |
| GGA (G) | **11** | **11** | **7** | **6** | **6** | **6** | **2** | **4** | **9** | 2 | **3** |
| GGC (G) | 2 | 2 | 1 | 1 | 1 | 1 | 1 | 4 | 0 | 1 | 1 |
| GGG (G) | 2 | 2 | 1 | 1 | 1 | 1 | 0 | 2 | 2 | 0 | 0 |
| GGT (G) | 3 | 3 | 3 | 3 | 3 | 3 | **2** | 2 | 4 | **11** | **3** |
| CAC (H) | 6 | 6 | 5 | 5 | 5 | 4 | 3 | 6 | 1 | 3 | 5 |
| CAT (H) | **17** | **17** | **18** | **20** | **18** | **16** | **10** | **16** | **12** | **13** | **17** |
| ATA (I) | 6 | 6 | 12 | 13 | 12 | 14 | 5 | 10 | 9 | 5 | 15 |
| ATC (I) | 6 | 6 | 4 | 4 | 4 | 3 | 1 | 3 | 2 | 5 | 4 |
| ATT (I) | **11** | **11** | **15** | **15** | **15** | **16** | **28** | **13** | **42** | **15** | **16** |
| AAA (K) | **28** | **28** | **41** | **38** | **41** | **47** | **325** | **75** | **67** | **41** | **45** |
| AAG (K) | 9 | 9 | 6 | 6 | 6 | 6 | 0 | 3 | 10 | 15 | 11 |
| CTA (L) | 1 | 1 | 1 | 1 | 1 | 1 | 0 | 1 | 1 | 1 | 2 |
| CTC (L) | 1 | 1 | 1 | 1 | 1 | 0 | 0 | 1 | 0 | 0 | 1 |
| CTG (L) | **3** | **3** | 2 | 2 | 2 | 1 | 0 | 2 | 0 | 1 | 1 |
| CTT (L) | 1 | 1 | 3 | 3 | 3 | 3 | 2 | 3 | 5 | 1 | 5 |
| TTA (L) | 1 | 1 | 3 | 3 | 3 | **9** | **8** | 5 | **18** | 8 | **9** |
| TTG (L) | **3** | **3** | **5** | **5** | **5** | 5 | 4 | **5** | 2 | **9** | 4 |
| ATG (M) | 18 | 18 | 19 | 20 | 18 | 16 | 10 | 16 | 22 | 20 | 16 |
| AAC (N) | 10 | 10 | 8 | 8 | 9 | 9 | 7 | 8 | 5 | 12 | 8 |
| AAT (N) | **27** | **27** | **31** | **31** | **30** | **32** | **28** | **27** | **43** | **35** | **33** |
| CCA (P) | **8** | **8** | **7** | **6** | **7** | **6** | **3** | **7** | 3 | **9** | **6** |
| CCC (P) | 1 | 1 | 1 | 1 | 1 | 1 | 0 | 2 | 0 | 1 | 0 |
| CCG (P) | 1 | 1 | 0 | 0 | 0 | 0 | 1 | 2 | 0 | 0 | 0 |
| CCT (P) | 3 | 3 | 3 | 3 | 3 | 3 | 1 | 2 | **6** | 4 | 5 |
| CAA (Q) | **27** | **27** | **24** | **23** | **23** | **21** | **16** | **21** | **18** | **13** | **22** |
| CAG (Q) | 8 | 8 | 7 | 7 | 7 | 6 | 2 | 7 | 2 | 2 | 6 |
| AGA (R) | **5** | **5** | **10** | **10** | **9** | **9** | **3** | **3** | **11** | **21** | **9** |
| AGG (R) | 3 | 3 | 2 | 2 | 2 | 2 | 0 | 1 | 2 | 4 | 2 |
| CGA (R) | 2 | 2 | 1 | 1 | 1 | 1 | 1 | 2 | 0 | 0 | 0 |
| CGC (R) | 0 | 0 | 0 | 0 | 0 | 0 | 0 | 1 | 0 | 0 | 0 |
| CGG (R) | 1 | 1 | 0 | 0 | 0 | 0 | 0 | 1 | 0 | 0 | 0 |
| CGT (R) | 1 | 1 | 0 | 0 | 1 | 1 | 1 | 1 | 0 | 2 | 0 |

**Table S3.** Summary statistics for beetle EST datasets.

| Species | *Chrysomela tremulae* | *Leptinotarsa decemlineata* | *Gastrophysa viridula* | *Callosobruchus maculatus* | *Sitophilus oryzae* | *Diabrotica virgifera* | *Ips pini* | *Dendroctonus ponderosae* | *Diaprepes abbreviatus* | *Hypothenemus hampei* |
| --- | --- | --- | --- | --- | --- | --- | --- | --- | --- | --- |
| Common name | Poplar leaf beetle | Colorado potato beetle | Green dock beetle | Cowpea weevil | Rice weevil | Western corn rootworm | Pine engraver | Mountain pine beetle | **Diaprepes root weevil** | Coffee berry borer |
| Food | Poplar leaves | Potato leaves | Dock leaves | Pulses/ beans | Rice grains | Maize roots | Pine xylem | Pine xylem | **Citrus roots** | Coffee beans |
| Tissue | Larval midgut | Larval midgut | Larval midgut | Whole larvae | Adult midgut | Larval midgut + adult head | Adult midgut | Adult midgut | Whole larvae | Larval midgut |
| Sequencing | 454 FLX | 454 Titanium | 454 Titanium | 454 Titanium | 454 Titanium | Sanger | Sanger | Sanger | Sanger | Sanger |
| Number of reads | 264,698 | 839,061 | 1,234,472 | 909,444 | 926,752 | 17,782 | 1,671 | 152,724 | 5,219 | 2,032 |
| Number of unique sequences after assembly | 10,910 | 21,692 | 20,817 | 32,584 | 22,989 | 7,686 | 618 | 17,528 | 1,987 | 854 |
| Number of contigs | - | - | - | - | - | 3,513 | 125 | 12,259 | 398 | 177 |
| Number of singletons | - | - | - | - | - | 4,173 | 493 | 5,269 | 1,589 | 677 |

**Table S4.** Families of beetle plant cell wall degrading enzymes identified in coleopteran-derived EST datasets.

| Family | **Chrysomelidae** | | | | | **Curculionidae** | | | | |
| --- | --- | --- | --- | --- | --- | --- | --- | --- | --- | --- |
| Species | *Chrysomela tremulae* | *Leptinotarsa decemlineata* | *Gastrophysa viridula* | *Callosobruchus maculatus* | *Diabrotica virgifera* | *Sitophilus oryzae* | *Ips pini* | *Dendroctonus ponderosae* | *Diaprepes abbreviatus* | *Hypothenemus hampei* |
| Number of unique sequences | 23,238 | 21,692 | 20,817 | 32,584 | 7,686 | 22,989 | 618 | 17,528 | 1,987 | 854 |
| **Cellulolytic enzymes:** | | | | | | | | | | |
| Endo-β-1,4-glucanase (GH45) | **2** | **7** | **1** | - | **4** | **5** | **6** | **9** | **5** | **2** |
| Cellulose 1,4-β-cellobiosidase  (GH48) | **2** | **3** | **3** | - | **1** | **2** | **3** | **6** | **1** | **2** |
| **Pectolytic enzymes:** | | | | | | | | | | |
| Endopolygalacturonase (GH28 subfamily A) | **9** | **10** | **7** | - | **3** | **7** | **2** | **19** | **4** | **2** |
| Endopolygalacturonase (GH28 subfamily B) | - | - | - | **7** | - | - | - | - | - | - |
| Pectin methylesterase (CE8) | - | - | - | - | - | **5** | **4** | **7** | **2** | - |
| Rhamnogalacturonate lyase (PL4) | - | - | - | - | - | - | **3** | **5** | - | - |
| **Other PCWDEs:** | | | | | | | | | | |
| β-mannanase (GH5 undefined subfamily) | - | - | **1** | **4** | - | - | - | - | - | - |
| β-mannanase (GH5 subfamily 7) | - | - | - | - | - | - | - | - | - | **2** |
| **Total** | **13** | **20** | **12** | **11** | **8** | **19** | **18** | **46** | **12** | **8** |

**Table S5.** cDNAs encoding beetle plant cell wall degrading enzymes identified from public databases. Genbank accession numbers are provided for each sequence.

| Super-family | **Chrysomeloidea** | | | | | **Curculionoidea** | | |
| --- | --- | --- | --- | --- | --- | --- | --- | --- |
| Family | **Chrysomelidae** | | **Cerambycidae** | | | **Curculionidae** | | |
| Species | *Phaedon cochleariae* | *Gastrophysa atrocyanea* | *Apriona germari* | *Psacothea hilaris* | *Oncideres albomarginata chamela* | *Otiorhynchus sulcatus* | *Hypothenemus hampei* | *Sitophilus oryzae* |
| Common name | Mustard leaf beetle | Unknown | Mulberry longicorn beetle | Yellow-spotted longicorn beetle | Unknown | Black vine weevil | Coffee berry borer | Rice weevil |
| **Cellulolytic enzymes:** | | | | | | | | |
| Endo-β-1,4-glucanase  (GH5 subfamily 2) | - | - | AAX18655.1 | BAB86867.1 | ADI24131.1 | - | - | - |
| Endo-β-1,4-glucanase (GH45) | CAA76931.1 | - | AAR22385.1  AAU44973.1 | - | ADI24132.1 | - | - | - |
| Cellulose 1,4-β-cellobiosidase  (GH48) | - | BAE94320.1  BAE94321.1 | - | - | - | CAH25542.1 | - | - |
| **Pectolytic enzymes:** | | | | | | | | |
| Endopolygalacturonase (GH28) | CAA76930.1 | - | - | - | - | - | - | AAG35693.1 |
| Pectin methylesterase (CE8) | - | - | - | - | - | - | - | AAW28928.1 |
| **Other PCW degrading enzymes:** | | | | | | | | |
| β-mannanase (GH5 subfamily7) | - | - | - | - | - | - | ACU52527.1 | - |
| Xylanase (GH11) | CAA76932.1 | - | - | - | - | - | - | - |

**Figure S1.** Predicted amino acid alignment of GH48 beetle enzymes. The amino acid sequence of the cellulose Cell48F from the bacterium *Clostridium cellulolyticum*, for which the crystal structure has been resolved, is used as a reference sequence (1). The catalytic residues (predicted from the *C cellulolyticum* sequence) are marked with arrows. Glu44 (numbering according to the *C. cellulolyticum* sequence) acts as the catalytic nucleophile/base, and Glu55 is the catalytic proton donor. Note that, in *G. viridula* Cellbio-3, the residue corresponding to the catalytic nucleophile/base is an Asp residue rather than a Glu.


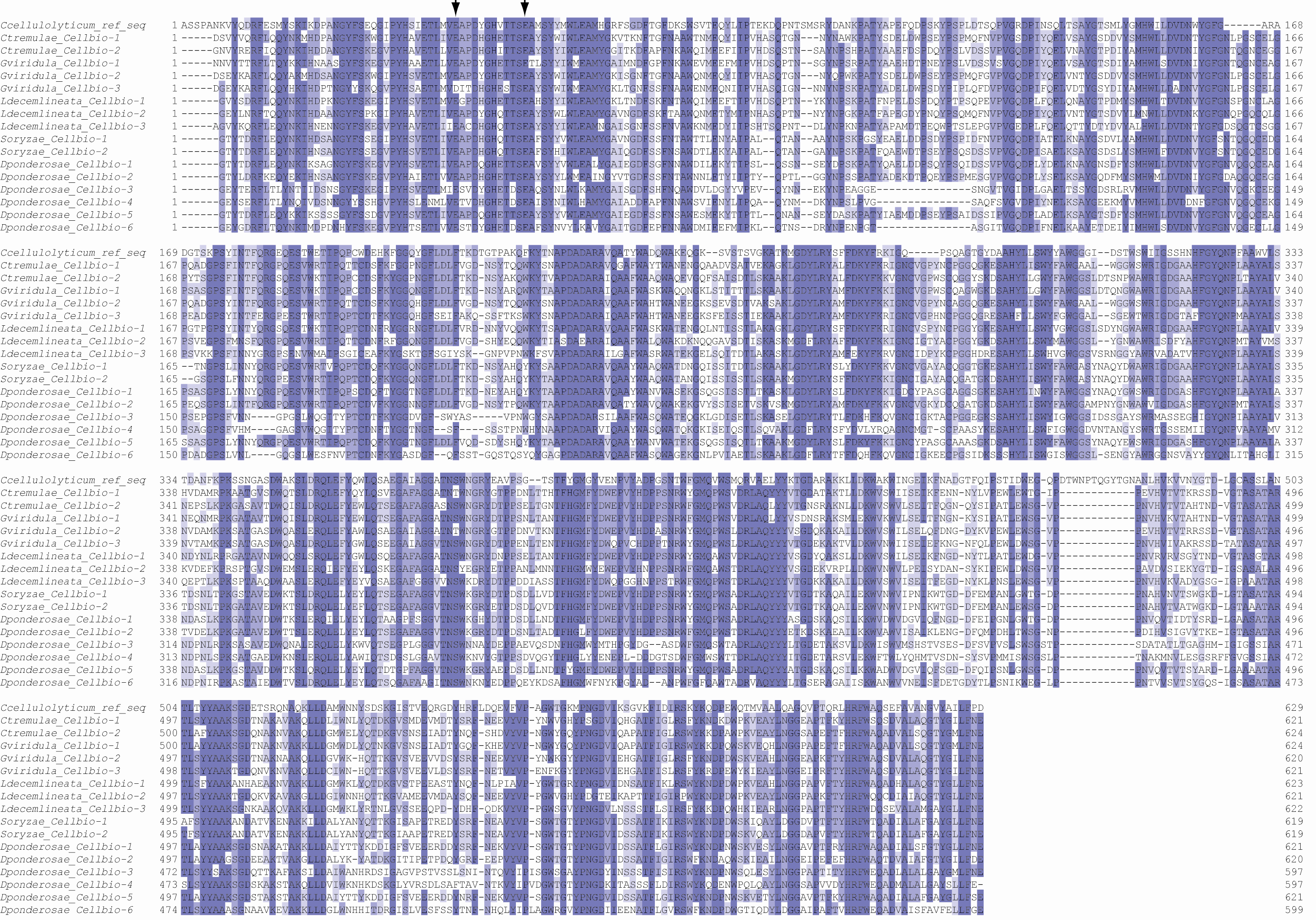


**Figure S2.** Predicted amino acid alignments of the beetle pectin methylesterases (CE8). The amino acid sequence of the pectin methylesterase from the fungus *Erwinia chrysanthemi*, for which the crystal structure has been resolved, is used as a reference sequence (2). The catalytic residues (predicted from the *E. chrysanthemi* sequence) are marked with arrows. Asp199 (numbering according to the *E. chrysanthemi* sequence) acts as the catalytic nucleophile/base, and Asp178 is the catalytic proton donor. Note that the catalytic nucleophile/base in *S. oryzae* Pectinesterase-5 is replaced by an Asn residue, suggesting a potential lack of catalytic activity for this protein.

**
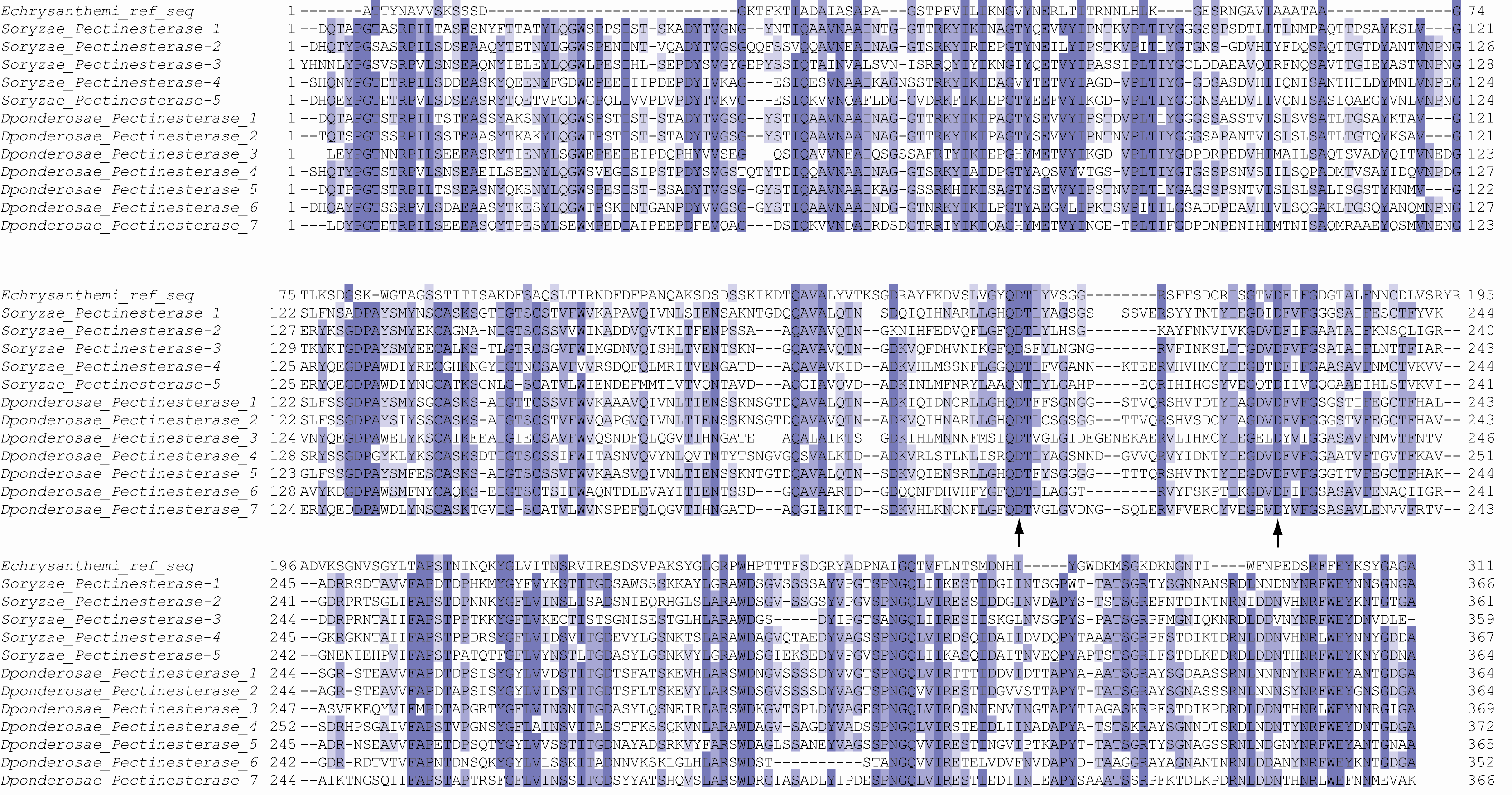
**

**Figure S3.** Predicted amino acid alignments of the beetle β-mannanase enzymes from a novel, unassigned, GH5 subfamily. The amino acid sequence of the β-mannanase from the Blue Mussel *Mytilus edulis*, for which the crystal structure has been resolved, is used as a reference sequence (3). The catalytic residues (predicted from *M. edulis*) are marked with arrows. Glu308 (numbering according to the *M. edulis* sequence) acts as the catalytic nucleophile/base, and Glu177 is the catalytic proton donor.


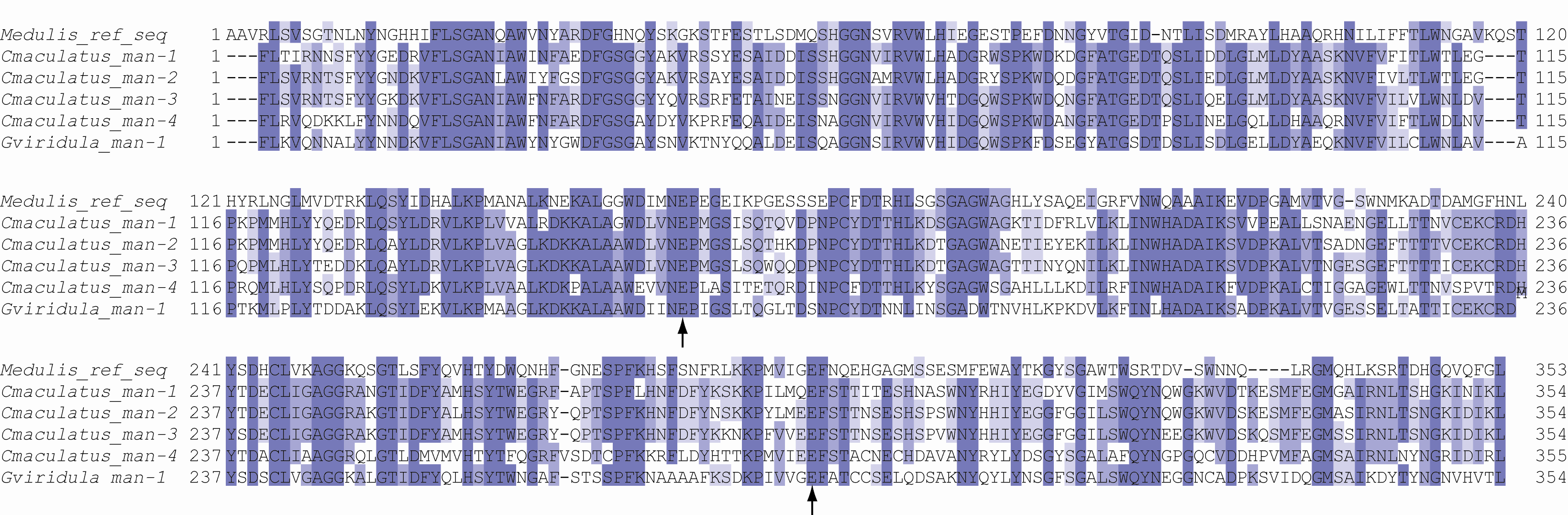


**Figure S4.** Predicted amino acid alignments of beetle-derived rhamnogalacturonate lyases (PL4). The amino acid sequence of RhiE, a rhamnose-induced protein from the plant pathogen *Erwinia chrysanthemi* harboring rhamnogalacturonate lyase activity on rhamnogalacturonan I, is used as a reference sequence (4).


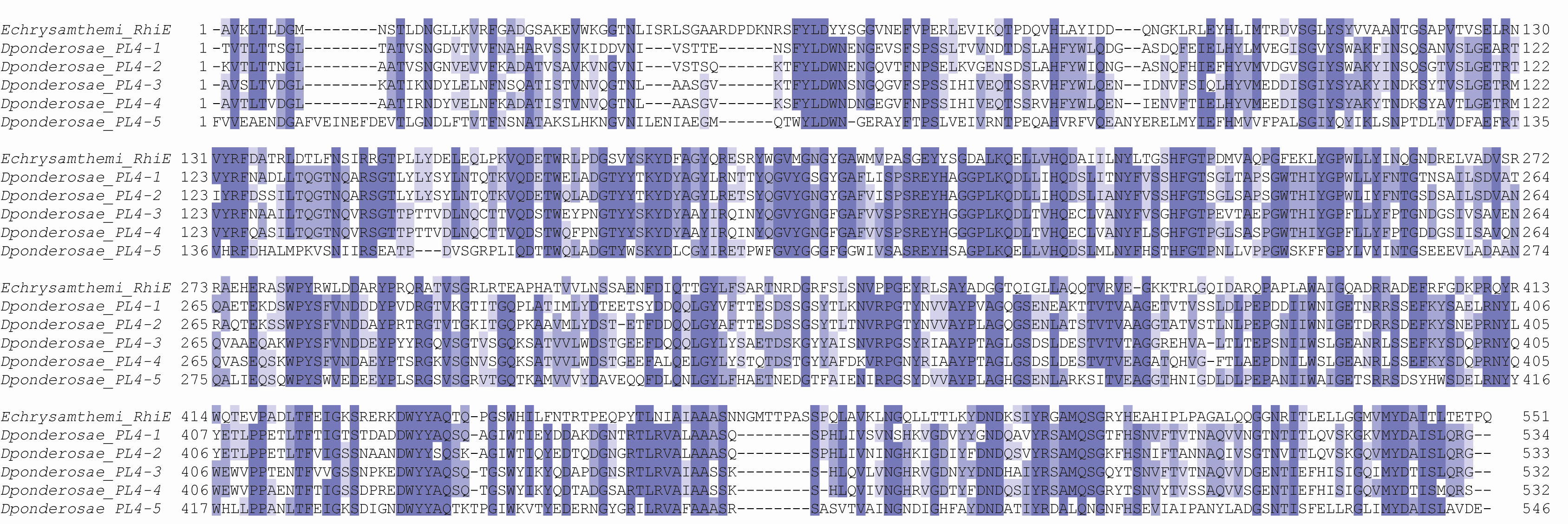


**References**

1. Parsiegla, G., Reverbel, C., Tardif, C., Driguez, H. & Haser, R. (2008) Structures of mutants of cellulase Cel48F of Clostridium cellulolyticum in complex with long hemithiocellooligosaccharides give rise to a new view of the substrate pathway during processive action. *J Mol Biol* **375,** 499-510.

2. Fries, M., Ihrig, J., Brocklehurst, K., Shevchik, V. E. & Pickersgill, R. W. (2007) Molecular basis of the activity of the phytopathogen pectin methylesterase. *Embo J* **26,** 3879-87.

3. Larsson, A. M., Anderson, L., Xu, B., Munoz, I. G., Uson, I., Janson, J. C., Stalbrand, H. & Stahlberg, J. (2006) Three-dimensional crystal structure and enzymic characterization of beta-mannanase Man5A from blue mussel *Mytilus edulis*. *J Mol Biol* **357,** 1500-10.

4. Laatu, M. & Condemine, G. (2003) Rhamnogalacturonate lyase RhiE is secreted by the out system in *Erwinia chrysanthemi*. *J Bacteriol* **185,** 1642-9.
